# Monitoring Glycolysis and Respiration Highlights Metabolic Inflexibility of *Cryptococcus neoformans*

**DOI:** 10.3390/pathogens9090684

**Published:** 2020-08-21

**Authors:** Sophie Lev, Cecilia Li, Desmarini Desmarini, David Liuwantara, Tania C. Sorrell, Wayne J. Hawthorne, Julianne T. Djordjevic

**Affiliations:** 1 Centre for Infectious Diseases and Microbiology, The Westmead Institute for Medical Research, Westmead, NSW 2145, Australia; levsophie@gmail.com (S.L.); Cecilia.Li@health.nsw.gov.au (C.L.); d.desmarini85@gmail.com (D.D.); tania.sorrell@sydney.edu.au (T.C.S.); 2 Sydney Medical School—Westmead, The University of Sydney, Westmead, NSW 2145, Australia; wayne.hawthorne@sydney.edu.au; 3 Marie Bashir Institute for Infectious Diseases and Biosecurity, University of Sydney, Sydney, NSW 2050, Australia; 4 Centre for Transplant and Renal Research, The Westmead Institute for Medical Research, Westmead, NSW 2145, Australia; liuwantara@gmail.com

**Keywords:** *Cryptococcus neoformans*, seahorse analyzer, ECAR, OCR, metabolic flux, glycolysis, respiration, human monocytes, *Candida albicans*

## Abstract

*Cryptococcus neoformans* is a human fungal pathogen that adapts its metabolism to cope with limited oxygen availability, nutrient deprivation and host phagocytes. To gain insight into cryptococcal metabolism, we optimized a protocol for the Seahorse Analyzer, which measures extracellular acidification rate (ECAR) and oxygen consumption rate (OCR) as indications of glycolytic and respiratory activities. In doing so we achieved effective immobilization of encapsulated cryptococci, established Rotenone/Antimycin A and 2-deoxyglucose as effective inhibitors of mitochondrial respiration and glycolysis, respectively, and optimized a microscopy-based method of data normalization. We applied the protocol to monitor metabolic changes in the pathogen alone and in co-culture with human blood-derived monocytes. We also compared metabolic flux in wild-type *C. neoformans,* its isogenic 5-PP-IP_5_/IP_7_-deficient metabolic mutant *kcs1*∆, the sister species of *C. neoformans, Cryptococcus deuterogattii/VGII,* and two other yeasts, *Saccharomyces cerevisiae* and *Candida albicans.* Our findings show that in contrast to monocytes and *C. albicans*, glycolysis and respiration are tightly coupled in *C. neoformans* and *C. deuterogattii,* as no compensatory increase in glycolysis occurred following inhibition of respiration. We also demonstrate that *kcs1*∆ has reduced metabolic activity that correlates with reduced mitochondrial function. Metabolic inflexibility in *C. neoformans* is therefore consistent with its obligate aerobe status and coincides with phagocyte tolerance of ingested cryptococcal cells.

## 1. Introduction

*Cryptococcus neoformans* is primarily an AIDS-related opportunistic fungal pathogen responsible for >180,000 deaths per year, mostly in sub-Saharan Africa [1]. A lung infection can establish in an immunocompromised host following inhalation of cryptococcal spores or small desiccated yeast cells, or after reactivation of a latent infection [2]. A robust lung infection commonly leads to fatal infection of the central nervous system (CNS) following transmission of the infecting cells from the lung via the blood or lymphatic system. *C. neoformans* grows both extracellularly and inside host phagocytes of the lung tissue and blood, with the latter facilitating dissemination to the brain [3,4,5], reviewed in [6]. Thus, an understanding of cryptococcal metabolism in both an extracellular and intracellular environment is required to fully understand pathogenicity.

*C. neoformans* is an obligate aerobe [7], which grows optimally under atmospheric levels of oxygen (approximately 21%) and relies heavily on mitochondrial respiration. In contrast, the growth rate of *Candida albicans* is unaffected by oxygen availability [7]. Growth of *C. neoformans* is therefore significantly compromised during the hypoxic conditions encountered during infection of the human host lung and the CNS [7,8,9] where infection-induced tissue damage and inflammation create an even more oxygen-restricted local microenvironment [10,11,12]. Functional mitochondria are therefore essential for survival of *C. neoformans* in the human host, with oxidative phosphorylation being upregulated to cope with hypoxia [8,9].

In addition to low oxygen, the human host lung is depleted of glucose. However, *C. neoformans* circumvents this by consuming carbon sources other than glucose [13,14,15], an adaptation mechanism requiring functional mitochondria. In contrast, glucose is a limiting nutrient for *C. albicans* [7,8,9]. We previously demonstrated that utilization of carbon sources other than glucose by *C. neoformans* depends on intracellular production of inositol pyrophosphate 5-PP-IP_5_/IP_7_ by the IP_6_ kinase Kcs1 and requires functional mitochondria. An IP_6_ kinase- (and hence 5-PP-IP_5_/IP_7_-) deficient metabolic mutant strain (*kcs1*Δ) therefore fails to grow on carbon sources other than glucose, establish a robust infection in the lungs and disseminate to the CNS in a mouse infection model [16].

To gain insight into the regulation of fungal metabolism, we sought the use of the Seahorse XFe24 Analyzer to monitor metabolic flux in cryptococcal cells. However, the original protocols were designed specifically for mammalian cells and therefore require optimization for fungal cells. The Seahorse Analyzer measures extracellular acidification rate (ECAR) and oxygen consumption rate (OCR). In mammalian cells, OCR is predominantly a measure of mitochondrial electron transport chain activity (i.e., respiration/oxidative phosphorylation), while ECAR is a combined measure of glycolysis and the tricarboxylic acid (TCA) cycle activity: pyruvate generated via glycolysis can be converted to lactate and excreted, leading to the acidification of the medium. Alternatively, pyruvate can enter the TCA cycle to be oxidized to CO_2_, which in an aqueous solution is hydrated to HCO_3_^−^ and H^+^ [17,18].

The Seahorse Analyzer can be used to measure cellular responses to modulators of glycolysis and oxidative phosphorylation and allows assessment of metabolic differences between cell types, the impact of genetic modification on metabolism and the identification of metabolic inhibitors as potential drug candidates. Due to differences in primary metabolism between fungal and mammalian cells and unique fungal surface properties that may impact cell adherence and permeability/efficacy of inhibitors (i.e., cell wall and polysaccharide capsule in *C. neoformans*), we have adapted and validated a Seahorse Analyzer protocol for *C. neoformans.* We applied the protocol to monitor metabolic changes in *C. neoformans* in co-culture with human blood-derived monocytes, and to compare metabolic flux in WT *C. neoformans* with a 5-PP-IP_5_/IP_7_-deficient metabolic mutant, its sister species *C. deuterogattii*/VGII [19,20], *S. cerevisiae* and *C. albicans.* The data obtained using the Seahorse Analyzer method demonstrates that, in contrast to other yeasts and mammalian cells, glycolysis and respiration are highly interdependent in *Cryptococcus,* which is consistent with its status as an obligate aerobe.

## 2. Results

### 2.1. Assay Optimization for C. neoformans

#### 2.1.1. Assay Medium and Cell Seeding Density

**Figure 1:**
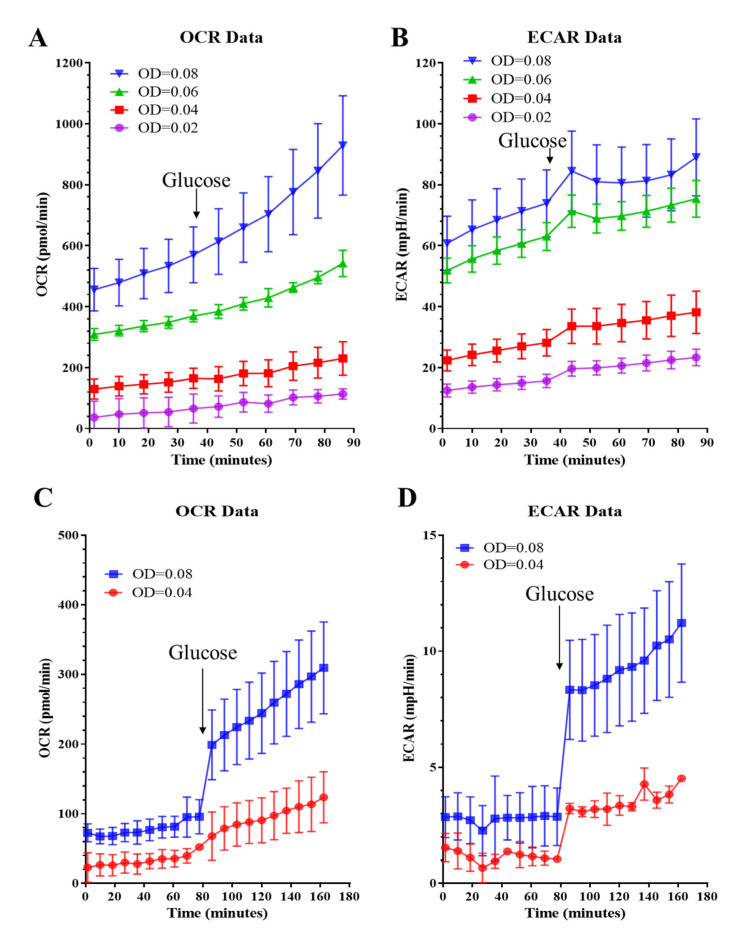
Optimization of the metabolic flux test for *C. neoformans* with respect to cell seeding density and assay medium. (**A**,**B**) OCR and ECAR drifts were observed when cryptococcal cells were equilibrated in assay medium containing 10 mM glucose. Subsequent addition of 20 mM glucose (arrow) further facilitated metabolic activity of the cells. Cells were seeded at different densities as indicated. (**C**,**D**) OCR and ECAR drifts were minimized in assay medium devoid of glucose. Addition of 20 mM glucose triggered significant respiratory activity. Assays were performed in 3–5 replicates and error bars represent SD. A cell seeding density of 0.02, 0.04, 0.06 and 0.08 equates to 1 × 10^6^, 2 × 10^6^, 3 × 10^6^ and 4 × 10^6^ cells per ml.

An optimal cell seeding density is crucial for obtaining consistent and meaningful metabolic profiles. To optimize the Seahorse XF Assay protocols for *C. neoformans*, we tested cell seeding densities ranging from OD_600_ = 0.02 to 0.08 in a standard Seahorse Assay medium. Although the starting OCR and ECAR values were within the recommended range for cell densities ranging between 0.04 and 0.08 (recommended OCR: 50–400 pmol/min, ECAR: 20–120 mpH/min), a significant and sustained increase in baseline OCR and ECAR was observed (Figure 1A,B). Glucose (10 mM) is a standard component of the Seahorse Assay medium when measuring the metabolic activity of mammalian cells. However, we found that a glucose concentration as low as 3 mM led to baseline drift in OCR and ECAR. This is most likely due to fungal cells having a higher growth (and hence glucose consumption) rate as compared to mammalian cells. Thus, glucose was omitted from the Seahorse Assay medium to keep baseline drift to a minimum and added later to boost metabolic activity prior to introducing the metabolic inhibitors (Figure 1C,D). Without glucose, the starting OCR and ECAR values were lower, but the OCR drift was minimized and the profiles were robust and consistent at cell seeding densities of 0.04–0.08. Addition of 20 mM glucose triggered a distinct increase in both OCR and ECAR (Figure 1C,D). Based on these observations, OD_600_ in the range of 0.04–0.08 was used for seeding *C. neoformans* cells.

#### 2.1.2. Mitochondrial Stress Test

**Figure 2:**
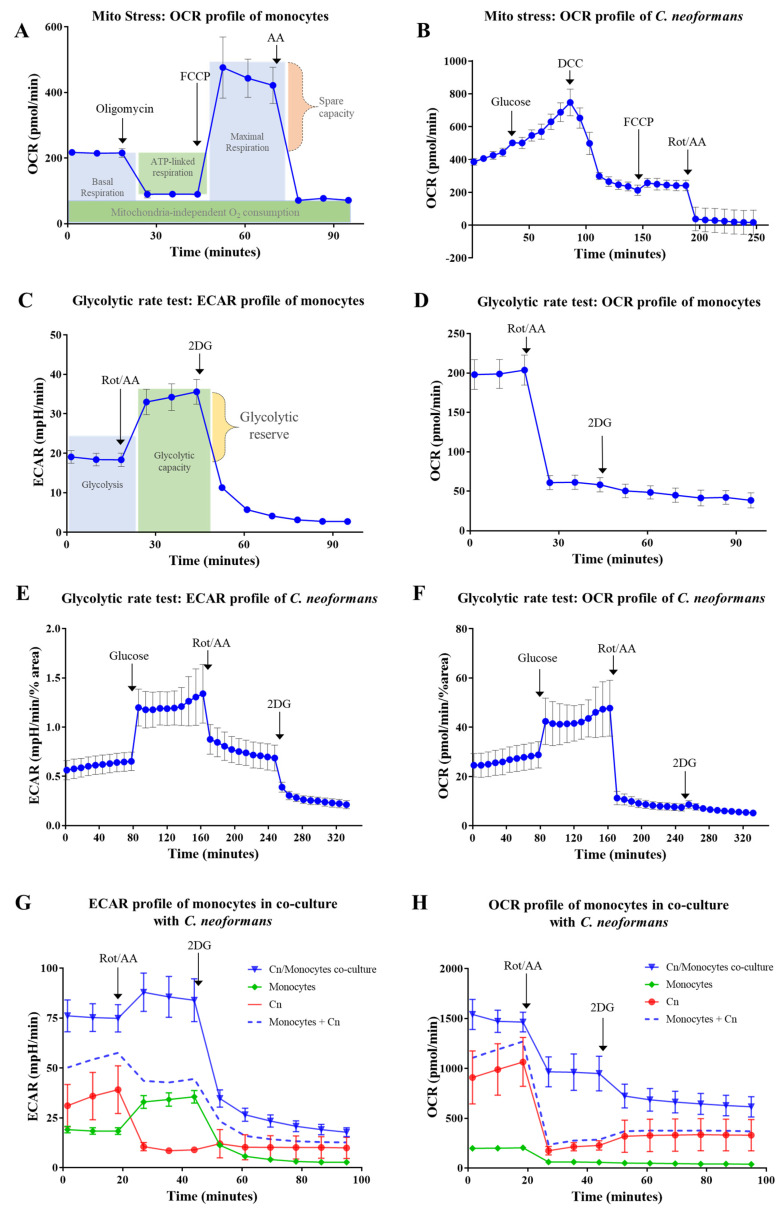
Metabolic profiles of mammalian cells (human monocytes) serve as a reference for customising a Seahorse assay protocol for *C. neoformans*. (**A**), OCR profile of human monocytes was generated following injection of the indicated compounds: mitochondrial ATP synthase inhibitor oligomycin (1 µM); uncoupler of oxidative phosphorylation FCCP—carbonylcyanide-4-trifluoromethoxyphenylhydrazone (0.6 µM); respiratory complex III inhibitor Antimycin A (10 µM). (**B**), OCR profile of *C. neoformans* was generated by injecting glucose (20 mM); mitochondrial ATP synthase inhibitor DCC—*N*,*N*′-Dicyclohexylcarbodiimide (50 µM); FCCP (5 µM); mixture of mitochondrial respiratory complex I and III inhibitors Rotenone and Antimycin A (Rot/AA, 50 µM). In this experiment, glucose is present in the basal medium and the data was not normalized. (**C**,**D**), ECAR and OCR profiles of monocytes generated by injection of Rot/AA (0.5 µM) and glycolysis inhibitor 2-deoxyglucose (2DG, 50 mM). (**E**,**F**), ECAR and OCR profiles of *C. neoformans* generated by the injection of glucose (20 mM); Rot/AA (50 µM); 2DG (100 mM). Glucose was absent from the basal medium and the profiles were normalized by the % area occupied by cells as described in Methods. (**G**,**H**), ECAR and OCR profiles of *C. neoformans*/monocyte co-culture. Profiles of each monoculture were obtained as a reference control. The sum ECAR and OCR profile of the monocultures is also shown by the dashed line to highlight metabolic differences unique to the co-culture. Inhibitors were injected as in (**C**). (**A**–**H**), Error bars represent standard deviation of a minimum of 3 replicates.

Firstly, we followed the mitochondrial stress test method established for mammalian cells. The main readout for this method is OCR, which is a measurement of oxygen consumption and a reflection of mitochondrial respiration associated with ATP production. When the mitochondrial ATP synthase (complex V) inhibitors such as oligomycin or *N*,*N*′-Dicyclohexylcarbodiimide (DCC) are injected, the OCR decreases. Subsequent injection of an uncoupler of oxidative phosphorylation, carbonylcyanide-4-trifluoromethoxyphenylhydrazone (FCCP) or carbonylcyanide-3-chlorophenylhydrazone (CCCP) disrupts mitochondrial membrane potential by collapsing the proton gradient and abolishing the link between ATP production and oxygen consumption. Oxygen is therefore consumed maximally by complex IV. The difference between maximal and basal respiration is termed spare respiratory capacity and is a measure of the ability of the cell to respond to increased energy demand. The last injection, which consists of a mixture of the complex I inhibitor, rotenone, and the complex III inhibitor, antimycin A (Rot/AA), inhibits mitochondrial respiration and enables measurement of mitochondria-independent oxygen consumption. Figure 2A demonstrates the mitochondrial stress test performed on CD14-positive monocytes isolated from human blood.

*C. neoformans* responds differently to metabolic inhibitors as compared to mammalian cells. For example, *C. neoformans* is insensitive to oligomycin [21,22]. We therefore investigated the use of an alternative ATP synthase (complex V) inhibitor, *N*,*N*′-Dicyclohexylcarbodiimide (DCC). A decline in oxygen consumption was observed following injection of DCC (50 µM), confirming that DCC is effective at inhibiting ATP synthase in *C. neoformans* (Figure 2B). Reduction in OCR following DCC injection is a measure of ATP-dependent respiration. In contrast to mammalian cells, subsequent injection of the uncoupler of oxidative phosphorylation, FCCP (5–100 μM), caused only a minor increase in OCR (Figure 2B), despite the growth of *C. neoformans* being inhibited by as little as 2 μM FCCP [23]. This suggests that despite the sensitivity of cryptococcal mitochondria to FCCP [24], mammalian and fungal cells respond differently to this uncoupling agent. Subsequent injection of the inhibitor mixture (Rot/AA) did, however, produce the expected outcome, almost abolishing oxygen consumption. This confirms that Rot/AA is an effective inhibitor of mitochondrial respiration in *C. neoformans.* Thus, consecutive use of ATP synthase inhibitors and an uncoupler of oxidative phosphorylation, as used for mammalian cells, is ineffective for assessing spare respiratory capacity in *C. neoformans*. However, respiratory inhibitors (Rot/AA) can be used for assessing the rate of glycolysis in *C. neoformans*.

#### 2.1.3. Glycolytic Rate Test

In the glycolytic rate test of mammalian cells (Figure 2C), basal ECAR measurements represent acidification of the medium due to the combined action of glycolysis and the TCA cycle. Addition of Rot/AA blocks mitochondrial respiration, as evidenced by the decrease in OCR following Rot/AA injection (Figure 2D). In response, to compensate for the decrease in ATP production, the rate of glycolysis is increased as the cells attempt to meet their energy demand. The end-product of glycolysis, pyruvate, is converted into lactate instead of entering the TCA cycle. Lactate is extruded from the cells, leading to an increase in ECAR, which reflects the fermentation capacity of the cells. Subsequent injection of 2-deoxyglucose (2DG) blocks the first two steps of glycolysis: 2DG is phosphorylated by hexokinase and accumulates in the cells to inhibit phosphoglucose isomerase in a competitive, and hexokinase in a non-competitive, manner [25,26]. Reduction of ECAR below the basal level reflects the inhibition of glycolysis by 2DG. This last step confirms that acidification of the medium following the inhibition of respiration is driven by glycolysis.

Based on our preliminary findings (Figure 1 and Figure 2B), we modified the glycolytic rate test used for mammalian cells for use in *C. neoformans.* This modified protocol involved: (1) equilibration in glucose-free medium; (2) injection of glucose to stimulate metabolic activity; (3) injection of Rot/AA to block cellular respiration; (4) injection of the early-stage glycolysis inhibitor, 2DG. Since *C. neoformans* is a non-fermenting yeast, production of other organic acids, most likely acetate rather than lactate, reflects its glycolytic rate (see Discussion). To support this conclusion, inhibition of glycolysis by 2DG in respiring cells that were not administered Rot/AA triggered an ~2-fold reduction in ECAR (data not shown). We then used this optimized protocol to assess ECAR and OCR profiles in *C. neoformans* (Figure 2E,F). Surprisingly, in contrast to mammalian cells, Rot/AA triggered a decrease in ECAR in *C. neoformans,* with OCR similarly dropping in both species (Figure 2E,F). This response suggests that despite the high demand for ATP, when oxidative phosphorylation is inhibited, glycolysis is also stalled, consistent with a limited capacity of *C. neoformans* to restore NAD^+^ via fermentation. Subsequent injection of 2DG caused a further drop in ECAR, consistent with inhibition of residual glycolysis (Figure 2E). The drop in ECAR induced by 2DG also signifies that the cells are metabolically active, and hence viable, following treatment with Rot/AA. No morphological changes indicative of cell stress or death were detected by microscopic examination at the end of the Assay (data not shown).

In summary, we have adapted the standard Glycolysis rate test to allow measurement of metabolic flux in *C. neoformans.* In doing so we have determined that *C. neoformans* responds differently to Rot/AA compared to mammalian cells due to tight coupling of glycolysis and oxidative phosphorylation/respiration.

### 2.2. Measuring Metabolic Activity of C. neoformans and Human Monocytes in Co-Culture

*C. neoformans* is rapidly internalized by phagocytes, which are a first-line immune defense mechanism. However, the internalized fungi can adapt and proliferate in phagolysosomes. Hence, blood-derived monocytes harboring viable fungal cells can provide a vehicle for cryptococcal dissemination to the brain [3,4,5], reviewed in [6]. In the case of bacterial and fungal infections, dramatic metabolic changes have been shown to occur in both the phagocyte and pathogen, which influence disease progression [27,28]. Infection polarizes phagocytes into M1 or M2 phenotype which is initiated by a profound switch in cellular metabolism: M1 (classical activation) is protective against *C. neoformans,* while M2 (alternative activation) is nonprotective [29]. M1 phagocytes have diminished TCA cycle activity and thus rely predominantly on glycolysis. This metabolic switch, characterized by increased glucose uptake and fermentation to lactate independently of mitochondrial function, is similar to the Warburg effect observed in tumors [30]. The Warburg effect is also elicited when phagocytes encounter bacteria and *C. albicans* [28,31,32,33]. In contrast, M2 phagocytes have a functional TCA cycle and are more dependent on oxidative phosphorylation. Monitoring metabolism of co-cultured *C. neoformans* and blood monocytes might provide insight into why *C. neoformans* is well tolerated inside phagocytes.

Having identified test conditions suitable for both mammalian and fungal cells, we co-cultured CD14-positive monocytes freshly isolated from human blood with *C. neoformans* and monitored their combined metabolic activity. We also monitored the metabolic activity of each monoculture as a reference. Recognition of the fungal cells by monocytes is followed by phagocytosis. The fungicidal capacity of monocytes towards cryptococcal cells is limited and internalized cryptococcal cells can survive and proliferate, eventually escaping with or without causing phagocyte death [34,35]. The latter is referred to as vomocytosis [36,37,38]. Starting from the early stages of pathogen recognition, host cells are likely to undergo metabolic changes that potentially limit fungal proliferation. We therefore started monitoring of the co-cultures within 2 h of the initial host–pathogen encounter.

The metabolic profiles in Figure 2G,H demonstrate that co-cultured cells (solid blue line) have higher metabolic activity (ECAR and OCR) than the individual cultures combined (dashed blue line). Higher ECAR activity in the co-culture is most likely attributable to increased glycolysis in monocytes, reminiscent of the Warburg effect, and is reflective of an elevated ATP demand by monocytes exposed to fungal cells. Addition of the respiratory inhibitors, Rot/AA, led to a marked increase in medium acidification by the co-cultured monocytes, which was greater than the combined ECAR of monocultures (dashed blue line). Thus, monocytes increase their glycolytic rate to compensate for the Rot/AA-inhibited respiration, while cryptococcal glycolysis is suppressed.

Oxygen consumption was also higher in the co-culture (solid blue line) than in the individual cultures combined (dashed blue line) (Figure 2H). This difference was especially prominent after the injection of Rot/AA, suggesting that oxygen, which is presumably consumed by the monocytes, is being used for respiration-independent processes such as production of reactive oxygen species and nitric oxide to minimize fungal proliferation.

### 2.3. Comparing the Metabolic Profiles of C. neoformans WT and the Mutant Strain, Kcs1*Δ*

**Figure 3:**
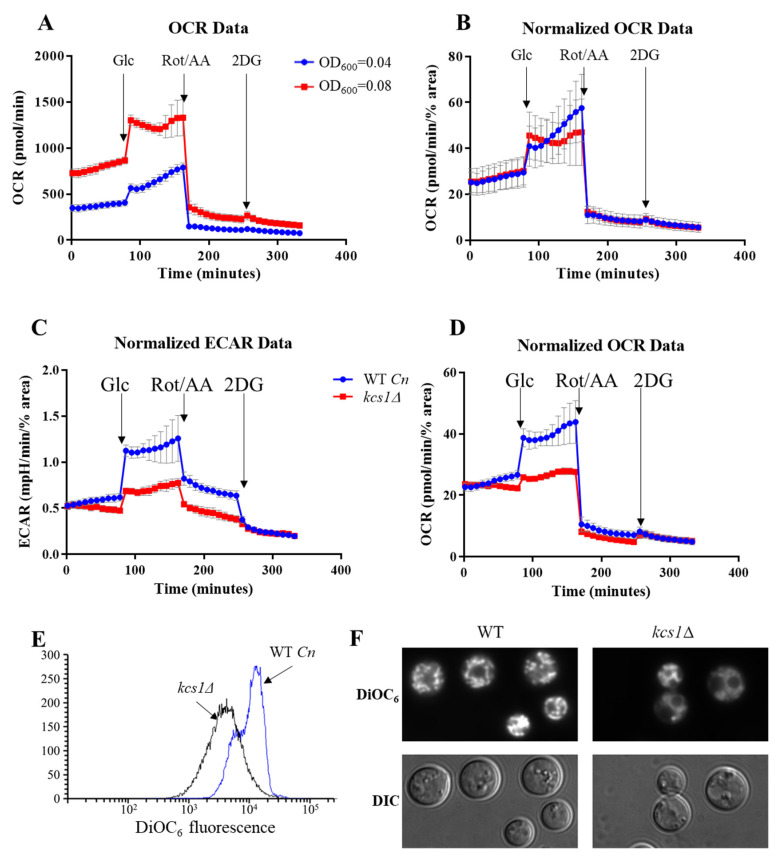
Metabolic profiles for *C. neoformans* WT and the *kcs1*Δ mutant. OCR profiles at two different cell seeding densities were obtained for WT *C. neoformans* following injection of the indicated compounds, before (**A**) and after (**B**) normalization by the cell-covered area method determined using Image J. (**C**,**D**), After data normalization, WT and the *kcs1*Δ mutant have different ECAR and OCR profiles, highlighting the presence of a metabolic defect caused by the absence of IP_7_. (**E**,**F**), DiOC_6_ staining of WT and *kcs1*Δ as assessed by flow cytometry (**E**) and fluorescence microscopy (**F**). Differential interference contrast (DIC) images are shown on the bottom panel.

When initially performing experiments with *C. neoformans,* particularly with the 5-PP-IP_5_-deficient *kcs1*∆ mutant, we observed a low rate of cell attachment to the poly-l-lysine coated wells. We speculated that this is attributable to the polysaccharide capsule, which is enlarged in the *kcs1*∆ mutant as compared to the WT [16]. The *kcs1*∆ mutant also has other unique surface properties including reduced mannoprotein exposure, which could affect its adherence [16]. We found that poor attachment of *C. neoformans*, particularly of the *kcs1*∆ mutant, could be alleviated by including a centrifugation step and by normalizing the OCR/ECAR data to percentage area of the Assay plate well occupied by cells, which was calculated using Image J (see Methods). Figure 3A,B demonstrates that differences in metabolic profiles due to differing seeding density are abolished in *C. neoformans* WT after normalization. This method was then applied to test metabolic flux in *kcs1*∆ cells.

Our previous work demonstrated that *kcs1*Δ mutant was unable to utilize carbon sources other than glucose and thus exhibited growth defects in vitro and in vivo in a mouse infection model. To further investigate this metabolic defect, we used our optimized protocol to compare metabolic flux in WT *C. neoformans* and the *kcs1*Δ mutant strain (Figure 3C,D). A seeding density of OD_600_ = 0.08 was used with the centrifugation step and cell adherence was normalized using Image J. The results show that basal ECAR and OCR are similar in both strains. However, following injection of glucose, the *kcs1*Δ strain did not increase glycolysis or respiration as efficiently as WT cells, even after an hour-long incubation with glucose. This is consistent with the previously reported metabolic defect in the *kcs1*Δ strain [16,39]. Despite the *kcs1*Δ strain having a lower metabolic rate, glycolysis and respiration were still tightly coupled. To further characterize the metabolic defect in *kcs1*Δ, we compared mitochondrial functionality in WT and *kcs1*Δ using the cationic dye, DIOC_6_. When used at low concentrations, DiOC_6_ accumulation in mitochondria correlates with the extent of mitochondrial membrane polarization [24,40]. DIOC_6_ was quantified by flow cytometry and visualized by fluorescence microscopy (Figure 3E,F, respectively). Reduced DIOC_6_ staining in the *kcs1*Δ strain is consistent with reduced oxidative phosphorylation.

### 2.4. Comparing the Metabolic Profiles of Three Yeast Species

**Figure 4:**
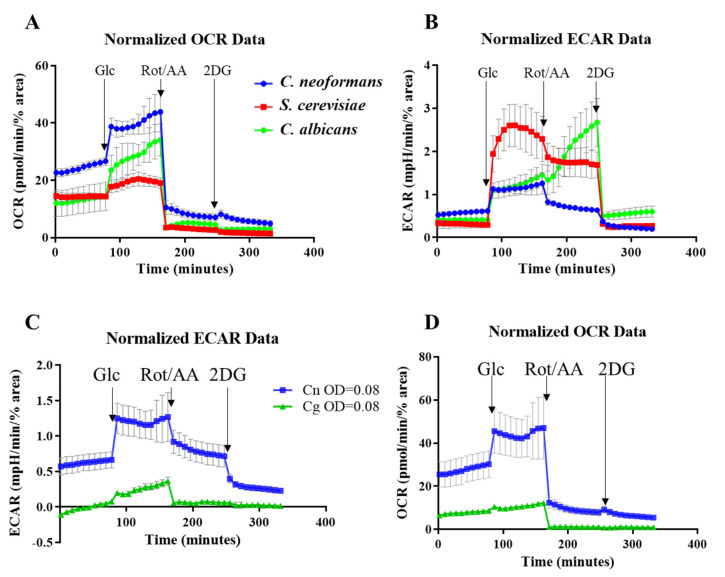
Comparison of OCR and ECAR profiles for *C. neoformans*, *S. verevisiae* and *C. albicans* (**A**,**B**), and for *C. neoformans* and *C. deuterogattii*/VGII (**C**,**D**). *C. neoformans*/*C. deuterogattii*/VGII have different profiles to *S. cerevisiae* and *C. albicans*, reflective of the varying capacity for fermentation. The following compounds were injected: Glc - glucose; Rot/AA – mixture of Rotenone and Antimycin A and 2DG – 2-deoxyglucose. Seeding density was OD_600_ = 0.08 and the results were normalized to the number of adhered cells determined using Image J. Error bars indicate standard deviation (n = 3).

Next, we used our method to compare metabolic flux in *C. neoformans* with that of *Candida albicans* and *Saccharomyces cerevisiae* using the same growth conditions (Figure 4A,B). All yeast cells were seeded at OD_600_ = 0.08 and the results were normalized as described above. Basal respiration was higher in *C. neoformans* as compared to the other two yeasts (see OCR profile in Figure 4A). All three species responded to the addition of glucose by increasing cellular respiration (elevated OCR). However, the increase was more pronounced in the pathogens (*C. neoformans* > *C. albicans* > *S. cerevisiae*). Rot/AA efficiently inhibited respiration in all species as reflected by the dramatic decrease in OCR. Addition of 2DG to block glycolysis had little effect on OCR in all three species.

For the ECAR profile, an increase in media acidification was observed for all species following the addition of glucose. However, unlike for OCR, the increase in ECAR was larger in *S. cerevisiae* than in the pathogens, reflective of its high fermentation capacity. Rot/AA treatment caused a decrease in ECAR in *S. cerevisiae* and *C. neoformans*, consistent with decreased CO_2_ production by TCA cycle. However, even after respiration was inhibited, the rate of glycolysis remained high in *S. cerevisiae* due to its high fermentation efficiency. In contrast to *C. neoformans* and *S. cerevisaie*, ECAR increased dramatically in *C. albicans* following Rot/AA treatment, consistent with a high rate of acetate production and an even more significant uncoupling of glycolysis and oxidative phosphorylation than that seen in *S. cerevisiae*. Our results showing how efficiently *C. albicans* undergoes a metabolic shift towards glycolysis is consistent with their ability to readily kill host macrophages by out-competing them in a glucose war [32]. Similarly, the inability of *C. neoformans* to favor glycolysis when mitochondrial function is perturbed coincides with its ability to survive inside host macrophages without killing them [34,35]. This may allow the host phagocyte to serve as a vehicle for dissemination of the infection to the CNS [3,5,6]. Addition of 2DG blocked glycolysis in all yeasts, returning ECAR values to a basal level (Figure 4B).

We also compared the metabolism of *C. neoformans* and *C. deuterogattii/VGII* (Figure 4C,D) and demonstrated that glycolysis and respiration are also tightly coupled in *C. deuterogattii/VGII.* However, *C. deuterogattii/VGII* had a lower basal metabolic activity and was less responsive to the addition of glucose.

## 3. Discussion

This is the first protocol optimized to monitor metabolic flux in pathogenic *Cryptococcus species* using the Seahorse Analyzer. The protocol involves measuring oxygen consumption and medium acidification by the fungal cells under nutrient-limited conditions, and following consecutive injection of glucose, respiratory inhibitors (Rot/AA) and the inhibitor of glycolysis (2DG).

**Figure 5:**
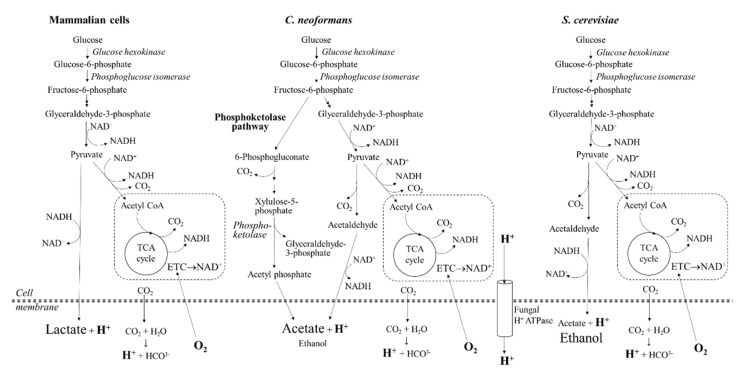
Schematic representation of the primary metabolic pathways in mammalian and fungal cells. In mammalian cells, glycolysis leads to the formation of pyruvate which is utilized in the TCA cycle and can also be converted to lactate. In the non-fermenting yeast, *C. neoformans*, acetate is produced via at least two routes: the phosphoketolase pathway (predicted based on the presence of the key enzymes) and pyruvate conversion to acetaldehyde. In the fermenting yeast *S. cerevisiae*, ethanol is the main fermentation product created even when glucose is abundant. Single dashed lines represent mitochondria where the electron transport chain (ETC) and tricarboxylic acid (TCA) cycle operate. Extracellular acidification (measured as ECAR) is caused by hydration of CO_2_ to form carbonic acid, extrusion of protons via plasma membrane H^+^-ATPase (fungi) and the production of lactate (mammalian cells) or acetate (fungi). Oxygen consumption rate (OCR) predominantly reflects mitochondrial respiration but can also reflect the production of reactive oxygen species (ROS) and reactive nitrogen species (RNS).

Although *C. neoformans* is a non-fermenting yeast, it releases significant amounts of acetate during host infection [41,42]. Although routes of acetate production have not been fully characterized in *C. neoformans,* several studies suggest that acetate is produced by the conversion of pyruvate to acetaldehyde and by the diversion of early intermediates of glycolysis into the phosphoketolase pathway [13,43] (Figure 5). The expression of several genes encoding enzymes involved in acetate production, including acetate kinase and pyruvate decarboxylase, is upregulated under hypoxic conditions and during host infection [9,13,43]. However, no NAD^+^ is regenerated via these pathways. In contrast to mammalian cells, very little lactate is produced by *C. neoformans* [41,42]. Another source of growth medium acidification by cryptococcal cells is the activity of P-type H^+^-ATPase. This proton pump maintains an electrochemical gradient across the fungal plasma membrane, which is essential for transport of multiple substrates. The function of H^+^-ATPase is replaced by Na^+^/K^+^ ATPase in mammalian cells. Thus, ECAR measurements in *C. neoformans* reflect excretion of acetate, carbonic acid and the export of protons by plasma membrane H^+^-ATPase.

Addition of glucose to the nutrient-limited Seahorse Assay medium boosts cryptococcal glycolysis, respiration and growth, as indicated by an increase in ECAR. Subsequent inhibition of mitochondrial oxidative phosphorylation by Rot/AA creates high demand for ATP, which cannot be fulfilled due to the inability of *C. neoformans* to regenerate NAD^+^ via fermentation. Consequently, glycolysis is largely stalled. Subsequent addition of the glycolysis inhibitor 2DG brings the rate of glycolysis (and ECAR) below the basal level.

We applied the protocol to monitor metabolic changes in *C. neoformans*, alone and in co-culture with human monocytes, and to compare metabolic flux of wild-type *C. neoformans* with its isogenic 5-PP-IP_5_/IP_7_-deficient mutant, which is impaired in central carbon metabolism, and with *S. cerevisiae* and *C. albicans.* In contrast to *C. neoformans*, Rot/AA inhibition of respiration in human monocytes, and the resulting reduction in ATP production, triggers an increase in glycolysis to compensate for the increased energy demand. Lactate is generated from pyruvate via glycolysis and is excreted, as reflected by an increase in ECAR (Figure 2C and Figure 5). In general, despite the dependence of mammalian cells on oxygen, their glycolytic and oxidative profiles can vary depending on the cell type and their immediate status (e.g., stress, disease, nutrient availability). For example, lymphocytes rely more heavily on oxidative phosphorylation, while neutrophils rely mainly on glycolysis (reviewed in [44]).

Numerous in-depth studies addressed metabolic changes that occur in monocytes and macrophages upon pathogen-mediated activation. Exposure of human monocytes to *C. albicans*, β-glucan and LPS triggers an increase in glycolysis, lactate secretion and ROS production [28,45]. Similarly, metabolic changes underpin polarization of macrophages, either to pro-inflammatory M1 or protective M2 [46]. M1 macrophages are highly glycolytic, have limited oxidative phosphorylation and increased flux via the pentose phosphate pathway, while M2 macrophages rely on the TCA cycle and oxidative phosphorylation. In fact, the TCA cycle becomes dysfunctional in M1 macrophages and respiration is largely impaired, rendering them insensitive to respiratory inhibitors [47]. In the case of cryptococcal infection, M1 polarization is associated with a more favorable outcome for the host. Basal ECAR and OCR profiles of the co-cultures demonstrate an increase in monocyte glycolysis suggesting a trend towards M1 polarization, which becomes more apparent when respiration is inhibited. This experiment demonstrates how tightly associated glycolysis and respiration are in *C. neoformans*, a weakness that could potentially be exploited by the use metabolic inhibitors that selectively target the pathogen, even when it is engulfed by host phagocytes.

Unlike *C. neoformans*, the non-pathogenic yeast, *S. cerevisiae,* has a high capacity for anaerobic glycolysis and fermentation, excreting ethanol as well as acetate and other fermentation products [48] (Figure 5). Even under aerobic conditions, when glucose is available, *S. cerevisiae* is actively fermenting, despite a lower ATP yield (otherwise known as the Crabtree effect) (reviewed in [49]). Another ascomycete, a pathogenic yeast commensal *C. albicans*, is predominantly aerobic, but has sufficient fermentation capacity to grow under anaerobic conditions [48]. Our data showing how efficiently *C. albicans* undergoes a metabolic shift towards glycolysis is consistent with its ability to readily kill host macrophages by out-competing them in a glucose war [32]. Similarly, the inability of *C. neoformans* to favor glycolysis when mitochondrial function is perturbed potentially makes it a less effective competitor for glucose, and coincides with its ability to survive inside host phagocytes without killing them [34,35]. This may allow the host phagocyte to provide a vehicle for dissemination of the infection to the CNS [3,4,5,6]. In support of this hypothesis, nitric oxide produced by activated macrophages inhibits the electron transport chain, and therefore macrophage respiration. It is possible that NO similarly affects internalized fungal cells or those in close proximity, thus suppressing their core metabolism.

Finally, using the Seahorse Analyzer method, we further investigated the metabolic defect previously reported in the *kcs1*∆ mutant [16]. The metabolic profiles obtained suggest that basal *kcs1*∆ metabolism is unaffected, but that the mutant has lost its ability to respond efficiently to the change in nutrient (glucose) availability. These data (namely reduced oxygen consumption in the presence of glucose) are consistent with reduced mitochondrial function as detected using a fluorescent dye which serves as an indicator of mitochondrial membrane polarization (Figure 3E,F). High-throughput gene expression data also suggest that the reduced glucose-responsiveness of *kcs1*∆ could be due to the reduced expression of hexose transporters, as the expression of genes encoding glycolytic enzyme is elevated in the *kcs1*∆ mutant [16].

In summary, we optimized a method to interrogate metabolic flux in *C. neoformans* and other yeast and used it to gain insight into core metabolism in fungal cells and its responses to chemical challenges and genetic intervention. Our protocol also provides a useful tool to analyze novel inhibitors of fungal-specific mitochondrial function, such as has been proposed by Kim, J.H. et al. [50]. Our experiments demonstrate that *C. neoformans* and *C. deuterogattii*/VGII are more highly reliant on mitochondrial function than other yeast, including *C. albicans,* consistent with previous descriptions as an obligate aerobe. The tighter coupling of glycolysis and oxidative phosphorylation in *Cryptococcus species* as compared to mammalian cells suggests that the development of fungus-specific metabolic inhibitors may offer an efficient treatment strategy for cryptococcosis.

## 4. Materials and Methods

### 4.1. Fungal Strains, Media and Reagents

Strains used in this study are *C. neoformans* (strain H99) and its isogenic mutant strain, *kcs1*Δ [16], *Candida albicans* (SN250) and *Saccharomyces cerevisiae* (BY4742) and *C. deuterogattii*/VGII (strain R265). Reagents include YPD broth, 0.01% poly-l-lysine, Cell-Tak (Corning), glucose and metabolic inhibitors (rotenone, antimycin A, 2-deoxyglucose, oligomycin, *N*,*N*′-Dicyclohexylcarbodiimide (DCC), carbonylcyanide-4-trifluoromethoxyphenylhydrazone (FCCP). Instruments include a 30 °C shaking incubator, a 37 °C stationary incubator and a Seahorse XFe24 Analyzer. Reagents associated with the Seahorse XFe24 Analyzer XFe24 include a FluxPak mini (contains 6 XFe24 sensor cartridges, 10 cell culture microplates and 500 mL of Calibrant solution) and Assay medium (Seahorse XF Base Medium supplemented with 2 mM L-glutamine and 5 mM HEPES pH 7.4).

### 4.2. Seahorse XFe24 Analyzer Setup and Optimization for Measuring Metabolism in C. neoformans

#### 4.2.1. Day before the Run

##### Sensor Cartridge Preparation

The cartridge was hydrated by pipetting 1 mL of XF Seahorse Calibrant solution into each well of the microplate in the cartridge pack. The cartridge was placed in a box containing a beaker filled with water to prevent drying out and incubated at 37 °C (no CO_2_) overnight. The wells of a cell culture microplate were coated with 0.01% poly-l-lysine by adding 50 µL of poly-l-lysine solution to each well and incubating for 1 h at room temperature. The poly-l-lysine solution was then removed, and the plate sealed with parafilm and stored at 4 °C.

##### Fungal Cell Preparation

Fungal cells from a freshly prepared agar plate were used to inoculate YPD broth and cultured overnight at 30 °C with shaking (250 rpm). YPD is the optimal growth medium as it does not support extensive capsule production in *C. neoformans*, which could potentially hinder cell attachment to the culture microplate.

#### 4.2.2. Day of the Run

##### CD14 Monocyte Preparation

PBMCs were isolated from whole blood using Ficoll-Paque Plus (GE Healthcare) as described previously [51], and CD14 monocytes were positively selected from PBMC suspension using CD14 MicroBeads (Miltenyi Biotec). Monocyte OCR and ECAR metabolic profiles were obtained following standard procedures for mammalian cells, using Assay medium containing 10 mM glucose and a cell density of 2.5 × 10^6^/mL with no confluency normalization required.

##### Fungal Cell Preparation

We established previously that there is a linear correlation between OD_600_ and number of cells per ml when the culture is diluted to an OD_600_ of 0.1–0.3. Thus for 0.1 OD_600_, the cell density is ~5 × 10^6^ cells/mL. Overnight fungal cultures were therefore diluted to an OD_600_ of 0.1–0.3 and this reading was used to obtain the final OD_600_ of the starter culture. Dilutions of between 0.02–0.08 were then used in the Assays. The overnight cultures were centrifuged, and the pellets were washed twice with water. The final pellet was resuspended in Assay medium at an OD_600_ of 0.4 Glucose-free Assay medium (Seahorse XF Base Medium) supplemented with 2 mM L-glutamine and 5 mM HEPES pH 7.4 (final pH adjusted to 7.4 with NaOH). Cell suspension (100 µL) was pipetted into each test well, leaving the background control wells empty. The microplate was centrifuged at 300× *g* for 5 min (acceleration 0, and deceleration 0) and then turned 180° and re-centrifuged to ensure efficient cell attachment and even dispersal of the cells for data consistency. Glucose-free Assay medium (400 µL) was slowly added to the test wells by pipetting through the side groove to achieve a final cell density of 0.08 (unless indicated otherwise). Assay medium (500 µL) was added to the background control wells. A photograph of the center of each well was taken at the lowest magnification (4× objective) for normalization (see “data normalization” below). The microplate was stored at 37 °C (atmospheric CO_2_) until ready to load to the Analyzer.

##### Sensor Cartridge Preparation

Compound stocks were prepared in fixed 10× concentrations in Assay medium. A 200 mM glucose stock and a 1M 2DG stock were prepared in Assay medium. Rot/AA was first prepared as a combined 2.5 mM stock in DMSO and then diluted in Assay medium to achieve a final stock concentration of 500 µM each. The injection ports of the sensor cartridge were loaded using the constant compound concentration/variable loading volume approach. For the glycolysis test, firstly, 56 µL of 200 mM glucose was added to Port A to achieve a final glucose concentration of 20 mM. Secondly, 62 µL of 500 µM Rot/AA was added to Port B to achieve a final concentration of 50 µM each. Lastly, 69 µL of 1 M 2DG was added to Port C to achieve a final concentration of 100 mM.

##### Assay Setup on Seahorse Analyzer

The Assay was set up using the Seahorse Wave software. The prepared sensor cartridge and cell culture microplate were loaded into the Seahorse XFe24 Analyzer as prompted by the instrument. Each stage of metabolic activity (the basal level prior to compound addition, then the activity after injection of each compound) was measured for up to 16 min (monocytes and co-cultures) or up to 1 h 20 min for *C. neoformans*.

### 4.3. Co-Culture Assay

CD14 positive human monocytes were prepared as described above. *C. neoformans* cells grown in YPD were opsonized with 10% human serum and anti-GXM 18B7 antibodies (10 µg/mL) (30 min, 37 °C). Fungal cells were pelleted at 4000 rpm for 3 min to remove as much serum as possible.

Culture and Seahorse Analyzer setup:

1. For host-pathogen co-culture samples, 100 µL of 250,000 CD14^+^ monocytes in Assay medium (with 10 mM glucose) were combined with 500,000 *C. neoformans* cells. The cells were mixed and plated on XF tissue culture plate pre-coated with Cell-Tak (Corning) following manufacturer’s instruction.
2. For cryptococcal control samples, 100 µL of Assay medium were added to cryptococcal cells. The cells were resuspended and plated on XF tissue culture plate.
3. For monocyte control samples, 100 µL of 250,000 CD14^+^ monocytes in Assay medium were plated on XF tissue culture plate.
4. The plate was incubated at 37 °C, 5% CO_2_ for 30 min.
5. To improve the attachment and provide even dispersal of the cells, the plate was centrifuged at 100× *g* for 1 min, deceleration 3. Then the plate was turned 180° and similarly centrifuged.
6. 400 µL Assay medium were slowly added to each well.
7. The plate was incubated at 37 °C without CO_2_ enrichment until ready to insert to the Analyzer (~1.5 h from the start of co-incubation).
8. Rot/AA 0.5 µM and 2DG 50 mM were injected into the plate wells during the course of the Assay.

### 4.4. Data Normalization for Fungal Cells

This step can be omitted if equal numbers of cells are added to the wells and cell attachment is close to 100%, as the readout from duplicate wells will be very similar. However, various factors such as the capsule of *C. neoformans* could affect cell attachment efficiency. To account for this, cell confluency was normalized using ImageJ (NCBI) as shown in point form below. Images were sharpened to improve cell separation and then “converted to mask” to create binary black and white images. A representative area of the image, or the entire image by default, was selected for particle analysis, using 3-infinity [in pixels] as the size. The percentage of the plate area covered by cells (% area) was calculated by the ImageJ software and used as a measure of cell confluency in each well. These data were pasted directly into the Wave normalization window.

- Process → Sharpen (improves separation between cells)
- Process → Binary → Convert to Mask (creates binary black and white image)
- Select representative area of the micrograph using buttons in the main ImageJ menu, or use the entire image by default
- Analyze → Analyze Particles (select Size as 3-infinity [in pixels]; tick “Summarize” option). If “%Area” is not displayed in the “Summary” window, tick “Area” option in the Analyze → Set Measurements window

### 4.5. DiOC_6_ Staining

DiOC_6_ staining was performed essentially as described in [24]. YPD overnight cultures of *C. neoformans* were adjusted to OD_600_ 0.5, and then stained with 3 µM DiOC_6_ in YPD for 30 min at room temperature in the dark. Cells were then washed once with water and resuspended in 1 mL water. Then 100 µL of the stained and washed cells was diluted in 500 µL water for flow cytometry, and the remaining cells were pelleted by centrifugation and mounted on a microscope slide for viewing by DeltaVision fluorescence microscopy.
